# Approach to Estimate the Phase Formation and the Mechanical Properties of Alloys Processed by Laser Powder Bed Fusion via Casting

**DOI:** 10.3390/ma15207266

**Published:** 2022-10-18

**Authors:** Uta Kühn, Jan Sander, Katharina Nicole Gabrysiak, Lars Giebeler, Konrad Kosiba, Stefan Pilz, Kai Neufeld, Anne Veronika Boehm, Julia Kristin Hufenbach

**Affiliations:** 1 Institute for Complex Materials, Leibniz IFW Dresden, Helmholtzstraße 20, 01069 Dresden, Germany; 2 Institute of Materials Science, TU Bergakademie Freiberg, Gustav-Zeuner-Str. 5, 09599 Freiberg, Germany

**Keywords:** additive manufacturing, centrifugal casting, laser powder bed fusion, mechanical properties, microstructure, tool steel

## Abstract

A high-performance tool steel with the nominal composition Fe85Cr4Mo8V2C1 (wt%) was processed by three different manufacturing techniques with rising cooling rates: conventional gravity casting, centrifugal casting and an additive manufacturing process, using laser powder bed fusion (LPBF). The resulting material of all processing routes reveals a microstructure, which is composed of martensite, austenite and carbides. However, comparing the size, the morphology and the weight fraction of the present phases, a significant difference of the gravity cast samples is evident, whereas the centrifugal cast material and the LPBF samples show certain commonalities leading finally to similar mechanical properties. This provides the opportunity to roughly estimate the mechanical properties of the material fabricated by LPBF. The major benefit arises from the required small material quantity and the low resources for the preparation of samples by centrifugal casting in comparison to the additive manufacturing process. Concluding, the present findings demonstrate the high attractiveness of centrifugal casting for the effective material screening and hence development of novel alloys adapted to LPBF-processing.

## 1. Introduction

Steels are known for their cost-efficient mass production combined with a broad and tunable variety of excellent properties leading to materials suitable for a broad range of structural applications [1,2]. In order to fabricate steel parts with a predestined geometry, multiple processing steps, such as casting, forming, heat treatment(s) and subtractive processing technologies, are required. Therefore, novel approaches for a more flexible, time- and resource-saving processing of products are explored. 

Additive manufacturing (AM) technologies such as the widely used laser powder bed fusion (LPBF), also known as selective laser melting (SLM), allow the direct fabrication of near-net-shaped components and, hence, significant savings in processing steps. Furthermore, waste material is reduced in comparison to subtractive processing. Due to the innovative layer-by-layer build up, parts with a great geometrical freedom and a high degree of individualization can be produced by such metal AM technologies [3].

However, there is a strong demand for LPBF-adapted alloys to fully exploit the potential of the AM process [4]. Currently, there is only a limited number of conventional alloys specifically qualified for metal AM such as LPBF (e.g., Al-Si10-Mg, Al-Si7-Mg0.6, 316L steel, Ti6Al-4V, CoCr28Mo6, CuNi2SiCr, CuCr1Zr) [5,6,7,8,9]. The development of novel alloys adapted to LPBF via the powder metallurgical route is expensive, laborious and energy- and resource-intensive, since firstly pre-alloyed powder must be produced for each composition mainly via gas atomization [10]. Sufficiently large amounts of powder are required for validation by LPBF, and powder changes in LPBF-devices are time-consuming, hence, not of high efficiency. Furthermore, controlling the successful LPBF fabrication of dense components with defined geometries is very challenging. Alloys must withstand a harsh processing environment characterized by high cooling rates of about 10^5^ K/s [11] of spatially localized molten pools and cyclic heat-treatment of solidified material due to heat extraction from overlying layers [12,13,14]. Moreover, the high cooling rates together with the cyclic short-term heat treatment lead to the evolution of complex and very fine-grained microstructures. The resulting mechanical properties of the additively manufactured material clearly differ from the corresponding conventionally cast or wrought counterparts [8,15]. Therefore, estimating the mechanical properties of LPBF-fabricated material by means of conventional gravity casting is hardly possible. However, an efficient alloy design for LPBF-suitable materials is required and could be realized via mimicking the high cooling rates during solidification. The only precondition for a successful realization of this concept is a sufficient castability of the alloy, potentially limited by a miscibility of the alloy components.

An approach based on melt spinning with cooling rates up to about 10^6^ Ks^−1^ was already demonstrated by Zhao et al. [16]. However, the appendant products were flakes or thin ribbons, which are mostly unsuitable for the characterization of the mechanical properties. Furthermore, similar approaches regarding the microstructural correlations between LPBF-processed samples prepared via rapid solidification techniques such as melt spinning and copper mold casting were also published [17].

This motivation is the starting point for the present work, which aims to provide a solution for approximating the mechanical properties of materials fabricated by LPBF. In this study, copper mold centrifugal casting with high cooling rates up to 10^4^ Ks^−1^ [18,19] was applied. In contrast to the powder-bed-based metal AM process, centrifugal casting is designed for the processing of small quantities to a high number of rapidly cooled samples. This effective casting method allows the rough estimation of the microstructure and mechanical properties of LPBF parts with an identical composition. In other words, centrifugal casting of small quantities permits the fast and efficient screening of alloys potentially suited to LPBF. 

For demonstrating the present approach, a high-performance tool steel was processed by gravity casting, centrifugal casting and LPBF. The nominal composition of Fe85Cr4Mo8V2C1 (wt%) was previously studied [20,21,22]. Conventional gravity casting was also employed to produce specimens which serve as reference. The microstructure and mechanical properties of the specimens produced by all three processing methods were studied as a function of the solidification rate. It was demonstrated that centrifugal casting sufficiently emulates the cooling conditions effective during LPBF, allowing the rough estimation of microstructure and mechanical properties of additively manufactured specimens.

## 2. Materials and Methods

An alloy with chemical composition Fe85Cr4Mo8V2C1 (wt%) was firstly produced by an induction melting process (Balzers, Germany) in a ceramic crucible (Al_2_O_3_) under argon atmosphere. The melt was cast into a copper mold (70 × 120 × 14 mm^3^) [21]. This ingot was used for the investigation of the material fabricated by gravity casting (GC) with solidification rates of about 10 to 70 Ks^−1^ [21]. Pieces of that ingot were processed at significantly higher solidification rates of about 10^4^ Ks^−1^ by centrifugal casting (CC) into a copper mold in purified argon atmosphere. The chamber of the centrifugal casting device (Linn High Therm, Hirschbach, Germany) contained a horizontal beam with a vacuum box in which the Cu-mold, the nozzle and the crucible were kept. After melting, the beam was rotated with a rate of 500 rpm, which applied a centrifugal force on the melt. The cylindrically shaped samples had a diameter of 3 mm and length of 70 mm. Pure elements were used for all melting processes.

Gas atomized powder with a particle size distribution of 15 to 63 µm was processed by LPBF in a SLM 250 HL device (SLM Solutions, Lübeck, Germany) equipped with a 400 W Yb:YAG laser. For the external gas atomization (TLS, Bitterfeld-Wolfen, Germany), cylindrical rods with 80 mm diameter and 200 mm height were produced by induction melting (as already described above and in previous studies [22]). 

Microstructural investigations were performed by scanning electron microscopy (SEM; Leo 1530 Gemini in combination with the SEM software SmartSEM, Zeiss, Jena, Germany), whereby the samples were finally polished with 0.25 μm diamond paste and deeply etched (87 mL Ethanol, 10 mL HNO_3_, 3 mL HCl, 5 g FeCl_3_*6 H_2_O). To localize the individual phases, electron backscatter diffraction (EBSD) point analysis was carried out (20 kV, Bruker eFlash HD, Berlin, Germany) after the additional preparation by vibratory polishing with MasterMet 2 (Buehler, Leinfelden-Echterdingen, Germany). The phase identification was based on the best fit solution, which was acquired and evaluated by the software Esprit 2.3 (Bruker, Germany).

For qualitative and quantitative phase analysis, X-ray diffraction measurements (XRD; Stadi P, Mo K*_α_*_1_ radiation, Mythen 1K detector, STOE, Darmstadt, Germany) were performed in transmission mode on samples with a thickness of about 80 μm. The recorded data were analyzed according to the Rietveld method [23] by using the Fullprof software (version 7.2, Grenoble, France) [24].

Quasi-static, room temperature compression tests (Instron 8562, Instron, Norwood, MA, USA) were conducted at a displacement rate of 10^−3^ s^−1^ using samples with a diameter of 3 mm and a height of 6 mm. Respectively, four compression stress–strain curves were analysed for determining the mean value and standard deviation of the 0.2 offset yield, ultimate strength and total strain. Furthermore, compression tests up to a total deformation of 10% were performed to study the phase transformation under mechanical loading by subsequent XRD analyses. In order to determine the microhardness, ten measurements with a load of 0.3 N were conducted (HMV–2, Shimadzu, Kyoto, Japan) for each steel sample processed with the three methods, and the appropriate standard deviation was calculated.

## 3. Results and Discussion

**Figure 1:**
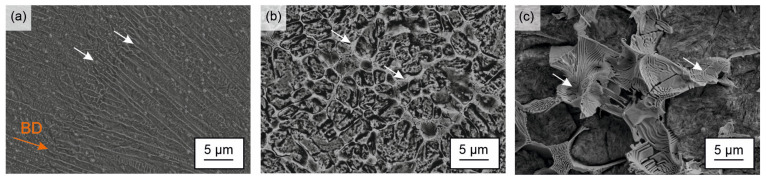
SEM images of the deep etched Fe85Cr4Mo8V2C1 steel processed by: (**a**) laser powder bed fusion (BD: building direction); (**b**) centrifugal casting; and (**c**) gravity casting. The carbide network is marked by arrows.

Figure 1a–c present SEM images of the deeply etched Fe85Cr4Mo8V2C1 steel processed by laser powder bed fusion, centrifugal casting and gravity casting. All samples synthesized by the three methods indicated the presence of carbides embedded in a matrix, which is assumed to be composed of martensite and austenite [22]. However, the microstructures showed a significant influence of the cooling rate. Regarding the carbides, a honeycombed network was notably pronounced in the sample produced by gravity casting but appeared also in the centrifugal cast sample just with finely graduated carbidic lamellas. By contrast, the carbides in the LPBF sample were even finer, round shaped and formed a discontinous network along the former austenite boundaries [25]. Furthermore, the SEM image displays elongated grains along the building direction. This microstructural feature is characteristic for LPBF-processed material and results from the directional solidification stemming from the major heat-extraction from the molten pools through the underlying material [14,22,25].

**Figure 2:**
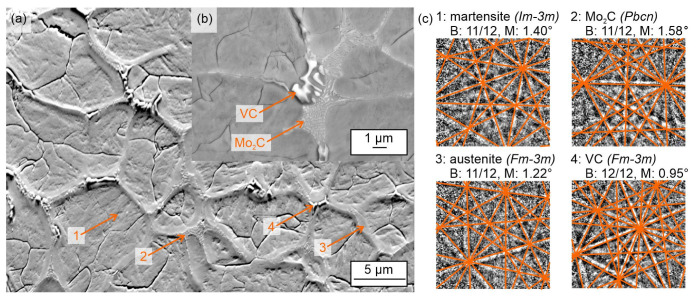
SEM and EBSD data of Fe85Cr4Mo8V2C1 after centrifugal casting: (**a**) fore scattered diffraction image showing the microstructure for the EBSD point analyses; (**b**) SEM image of the carbide morphology; and (**c**) experimental Kikuchi pattern of the point analyses with best fit solution of the simulated pattern as overlay. For the evaluation of the fit, the number of matched bands (B) and the band mismatch (M) are given.

Figure 2a,c present an orientation contrast image (fore scattered diffraction, FSD) and Kikuchi patterns (aquired by point EBSD) of the alloy after centrifugal casting. The carbide network consists of fine, lamellar Mo_2_C carbides as well as bench- or coral-like VC carbides. These two different kinds of carbide morphologies were already reported in a previous study for the GC state [21] as well as by Hwang et al. [26] and by Luan et al. [27] for the CC state, although they are clearly finer in this study (Figure 2b). The phases were investigated by EBSD based on crystal structure parameters detected by XRD (see structural data in Table 1). Two different carbides could be confirmed by EBSD; representative Kikuchi patterns and respective fits with simulated patterns are presented in Figure 2c. Furthermore, austenite and martensite could be detected (Figure 2c) in accordance with the other samples after GC and LPBF.

**Figure 3:**
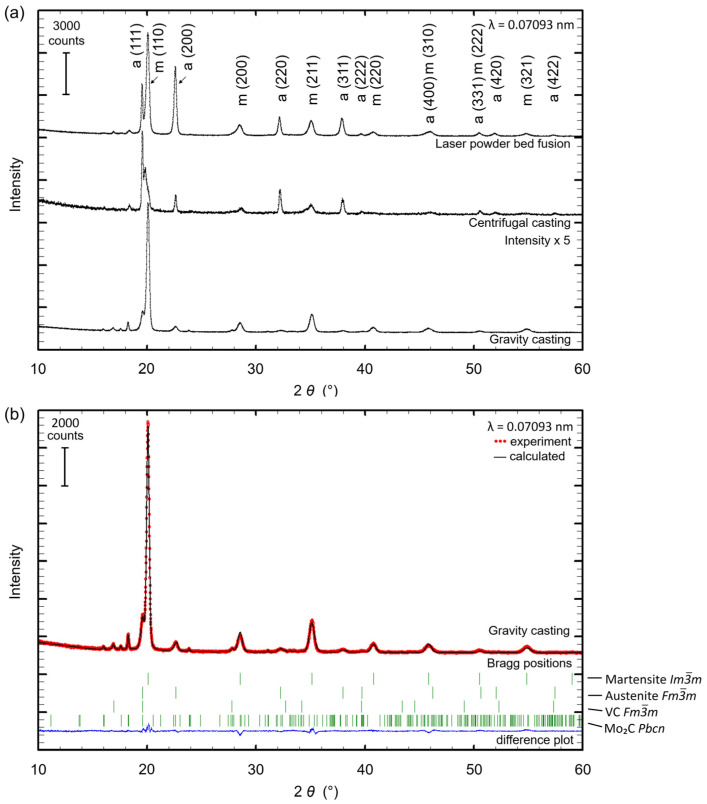
(**a**) XRD patterns for Fe85Cr4Mo8V2C1 processed by laser powder bed fusion, centrifugal casting and gravity casting. For reasons of clarity, only austenite and martensite are indexed; (**b**) Rietveld plot of the sample produced by gravity casting with the contributing phases (from top to bottom) martensite, austenite, VC and Mo_2_C as symbolized by the vertical, green Bragg position markers. The difference plot at the bottom of the graph (continuous, blue line) displays the quality of the calculated fit (continuous, black line) in comparison with the observed data (dotted, red line).

Figure 3a presents the XRD patterns of samples processed by laser powder bed fusion, centrifugal and gravity casting. Table 1 lists the phases, the respective content and the lattice parameters determined by the Rietveld analyses as exemplarily shown for a sample prepared by gravity casting (Figure 3b). Due to the different manufacturing technologies, the individual cooling rates strongly affected the volume fraction of the phases. The phase content of retained austenite with space group (SG) *Fm*-3*m* [28] increased from 16 wt% to 38 wt% with increasing cooling rates, whereas the martensite (SG *Im*-3*m*) [29] content decreased. One possible explanation for the origin of this effect is the limited carbon diffusion at high cooling rates. Carbon remained slightly more concentrated in the austenite eventually leading to a certain stabilization of this phase. Another explanation is related to the increased undercooling resulting from higher cooling rates effective during solidification. Stronger undercooling drives the nucleation of a higher density of austenitic grains [30,31,32,33]. Due to a smaller grain size, a lower martensite start temperature follows, resulting in the formation of less martensite [34,35,36,37]. This phenomenon is known as mechanical stabilization of the austenite [38]. Low fractions of the two carbide phases—cubic VC with SG *Fm*-3*m* [39] and orthorhombic Mo_2_C with SG *Pbcn* [40]—were observed for all samples and reduced with increasing cooling rate from 10 wt% for gravity casting to 5 wt% for LPBF, due to suppressed carbon diffusion at high cooling rates. It is noted that preferred orientations and strain contribute with differing influence as indicated by unusual reflection intensity accentuation and reflection broadening at higher 2*θ* angles where faster cooling occurred. It is additionally noted that the used carbide structure models of VC and Mo_2_C should be understood as type-like structure models. Hence, Cr can substitute V and Mo to a small extent [25,41]. Additionally, for the fits of the X-ray patterns with fast cooling rates, a second martensite phase was necessary to at least enable a sufficiently calculated pattern. Here, an inhomogeneous distribution of elements is assumed as the reason initiating the corresponding changes in the lattice parameters into a larger and a smaller portion compared to the lattice parameters found in the gravity cast sample (Table 1).

The LPBF sample showed a second martensite phase but the fit was sufficient with one martensite phase as the reflection splitting was less dominant compared to the centrifugal casting sample. However, the most important aspect of the investigation was found in the similar or even equal phase contents (martensite: 59/57 wt%; austenite: 36/38 wt%; carbides: 5/5 wt%) for the samples prepared by centrifugal casting and laser powder bed fusion, respectively. 

**Figure 4:**
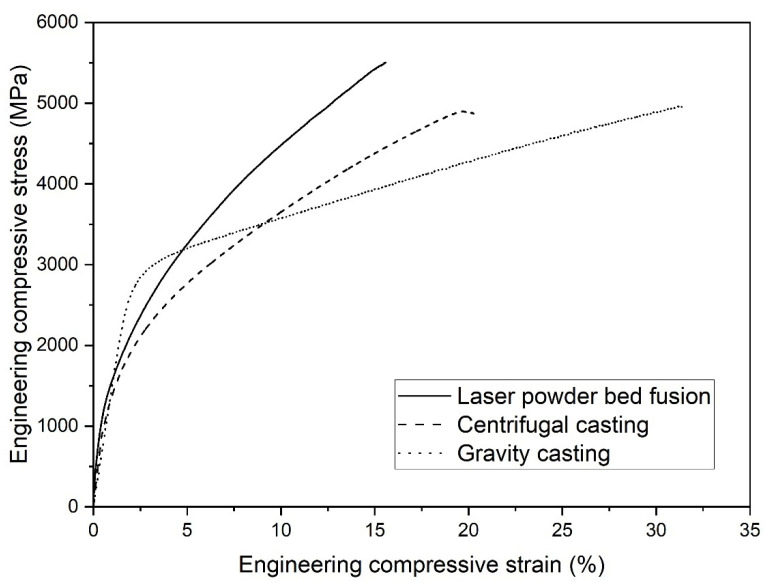
Representative room temperature engineering compression stress–strain curves of the Fe85Cr4Mo8V2C1 processed by gravity casting, centrifugal casting and laser powder bed fusion.

The results of the microstructural characterization of the different sample states (GC, CC, LPBF) are also reflected in the mechanical behavior under compressive load (Figure 4, Table 2). Figure 4 displays representative engineering stress–strain curves of the three conditions, GC, CC and LPBF, whereby the curve of the GC sample subjected to the lowest cooling rate particularly distinguishes from the CC and LPBF specimens. The GC sample contains the highest amount of martensite (74 wt%) and carbides (10 wt%) leading to the highest yield strength among the samples (Table 2).

In contrast, the mechanical behavior of the CC sample enables us to draw conclusions about the behavior of the LPBF specimen, despite the differences in size and morphology of the phases. The similar phase contents in both conditions result in a similar compressive yield strength and total strain of the CC and the LPBF within the scope of the standard deviation (Table 2). The reduced ductility of the CC and LPBF specimens could be traced back to a significantly higher porosity in comparison to the GC samples, since those defects trigger crack initiation and propagation and promote early failure. Especially the LPBF samples suffer from lack of fusion pores generated by insufficient layer bonding in the printing process (not shown here). Furthermore, due to the higher cooling rates and large temperature gradients in the CC and LPBF process, a higher degree of residual stresses is assumed in the respective samples [42] in comparison to the GC specimens, which also affects the deformation and failure behavior.

Furthermore, the CC and LPBF specimens show engineering compressive stress–strain curves with a high degree of work hardening, which can be attributed to, among others, to the transformation of retained austenite into martensite. This phenomenon is known as transformation-induced plasticity (TRIP) and is characteristic for this type of high-carbon steels [41,43,44]. In order to provide evidence regarding the TRIP effect of the investigated alloy, compression tests up to a strain level of 10% were conducted, and subsequently the mass fractions of retained austenite and martensite were determined by XRD. As expected, the sample prepared by conventional gravity casting shows just a slight increase of the martensite mass fraction of 10 wt% during deformation since the initial value of martensite content is comparatively high.

In contrast, the samples fabricated by CC and LPBF contain a high amount of retained austenite and reveal an increase of martensite of 27 wt% and 23 wt%, respectively. This result underscores once more the analogous mechanical performance of the samples prepared by these manufacturing routes. 

Regarding the microhardness, the LPBF and the CC samples present with an average of 900 HV0.3 and 845 HV0.3, respectively, clearly higher hardness values compared to the GC sample (710 HV0.3). This can be attributed to the significantly finer microstructure (Figure 1) but is, at first glance, contrary to the lower offset yield strength of both conditions. However, it is known from the literature that the indenter of the hardness tester induces shear stress and deforms the material plastically, leading to a direct austenite into martensite transformation already during the indentation process [45,46]. Consequently, the hardness of the material with high contents of metastable retained austenite (36/38 wt%) is shifted to higher values because of the TRIP effect, and the real hardness is hardly determinable with the usual testing method. Indeed, the microstructure of the GC sample presents also an austenitic phase, but the content of 16 wt% is significantly lower compared to LPBF and CC. Therefore, the TRIP effect is less pronounced during mechanical loading for this condition.

## 4. Conclusions

In this study, a high-strength Fe85Cr4Mo8V2C1 steel was successfully produced by three manufacturing methods characterized by increasing cooling rates effective during solidification: gravity casting, centrifugal casting and laser powder bed fusion. 

For all processing routes, a microstructure composed of martensite, austenite and complex carbides was investigated by electron microscopy and X-ray diffraction. The gravity cast material reveals a significant coarser microstructure and different weight fractions of the phases compared to the samples manufactured by the other methods, which also results in deviant mechanical behavior. 

However, by centrifugal casting, a method was provided, which enables us to estimate the phase contents and, hence, to a certain extent, the mechanical properties of samples manufactured by LPBF although slightly lower cooling rates are obtained by this method. Thus, the average 0.2% offset yield strengths under compressive load of samples processed by centrifugal casting and laser powder bed fusion are in the same range considering the standard deviation. Furthermore, a similar microhardness could be obtained for these two processing states. 

Concluding, the centrifugal casting technology is a very effective, resource-efficient and sustainable method for the development of novel alloys adapted to additive manufacturing processing such as laser powder bed fusion.

## Figures and Tables

**Table 1:** Structure models, lattice parameters and phase contents of martensite, austenite and the carbides in Fe85Cr4Mo8V2C1 determined by Rietveld analyses of X-ray diffraction data.

Sample	Phase	Space Group	*a*/nm	*b*/nm	*c*/nm	*V*/nm^3^	Content/ wt%
Gravity casting	Fe	*Im-3m*	0.28792(3)			0.023867(8)	74
	Fe0.94C0.06	*Fm-3m*	0.3615(2)			0.04723(8)	16
	VC	*Fm-3m*	0.4181(3)			0.07307(15)	6
	Mo_2_C	*Pbcn*	0.4641(3)	0.5903(3)	0.5088(3)	0.1394(2)	4
Centrifugal casting	Fe	*Im-3m*	0.29157(8)			0.02479(2)	33
	Fe	*Im-3m*	0.28812(12)			0.02392(3)	26
	Fe0.94C0.06	*Fm-3m*	0.36138(2)			0.047194(9)	36
	VC	*Fm-3m*	0.4178(-)			0.07295(-)	2
	Mo_2_C	*Pbcn*	0.4599(7)	0.5863(13)	0.506(2)	0.1364(10)	3
Laser powder bed fusion	Fe	*Im-3m*	0.2885(3)			0.02401(7)	57
	Fe0.94C0.06	*Fm-3m*	0.3625(3)			0.0476(1)	38
	VC	*Fm-3m*	0.4181(7)			0.0731(3)	3
	Mo_2_C	*Pbcn*	0.462(3)	0.589(4)	0.505(4)	0.138(3)	2

**Table 2:** Engineering values of room temperature compression tests and microhardness for Fe85Cr4Mo8V2C1 samples processed by gravity and centrifugal casting as well as by laser powder bed fusion.

Processing Route for FeCrMoVC	Yield Strength, 0.2% Offset/MPa	Ultimate Compressive Strength/MPa	Total Compressive Strain/%	Microhardness HV0.3
Gravity casting	2494 ± 11	4909 ± 117	31.5 ± 0.6	710 ± 34
Centrifugal casting	1345 ± 86	4688 ± 390	18.6 ± 2.4	845 ± 19
Laser Powder Bed Fusion	1338 ± 44	5326 ± 171	15.6 ± 1.0	900 ± 12

## Data Availability

Derived data supporting the findings of this study are available from the corresponding author upon request.
